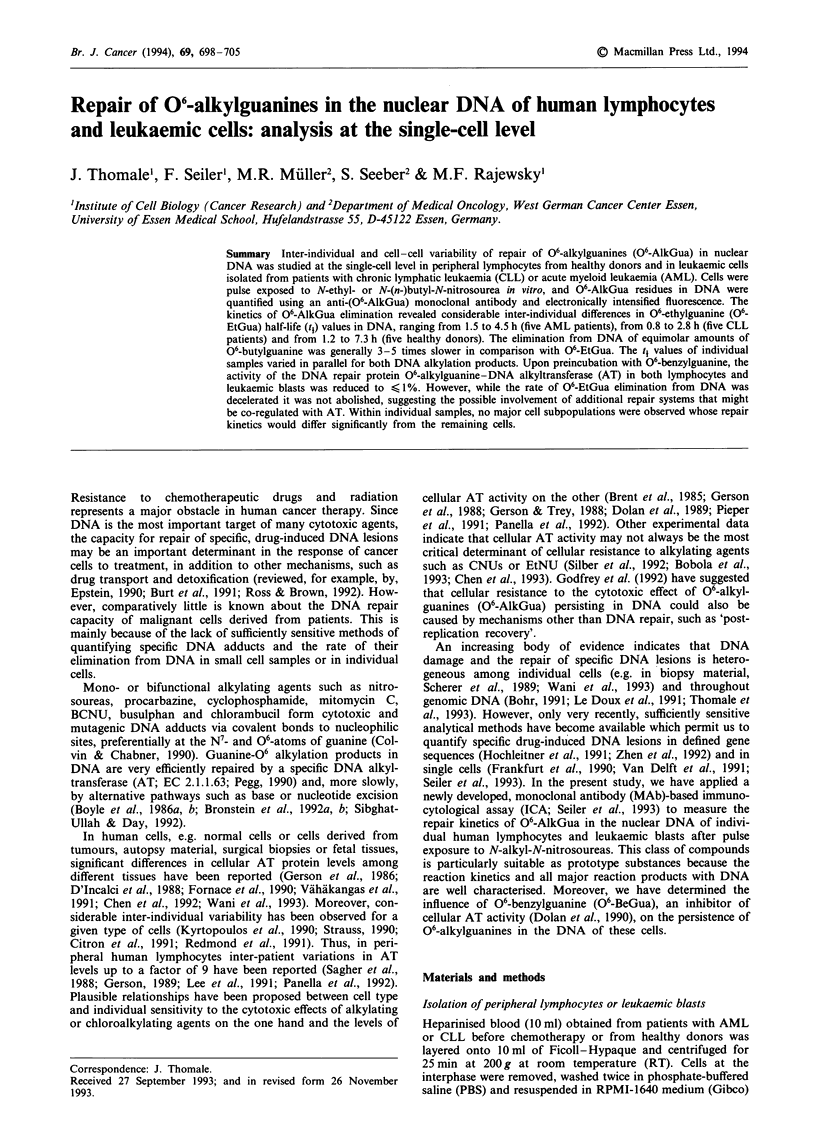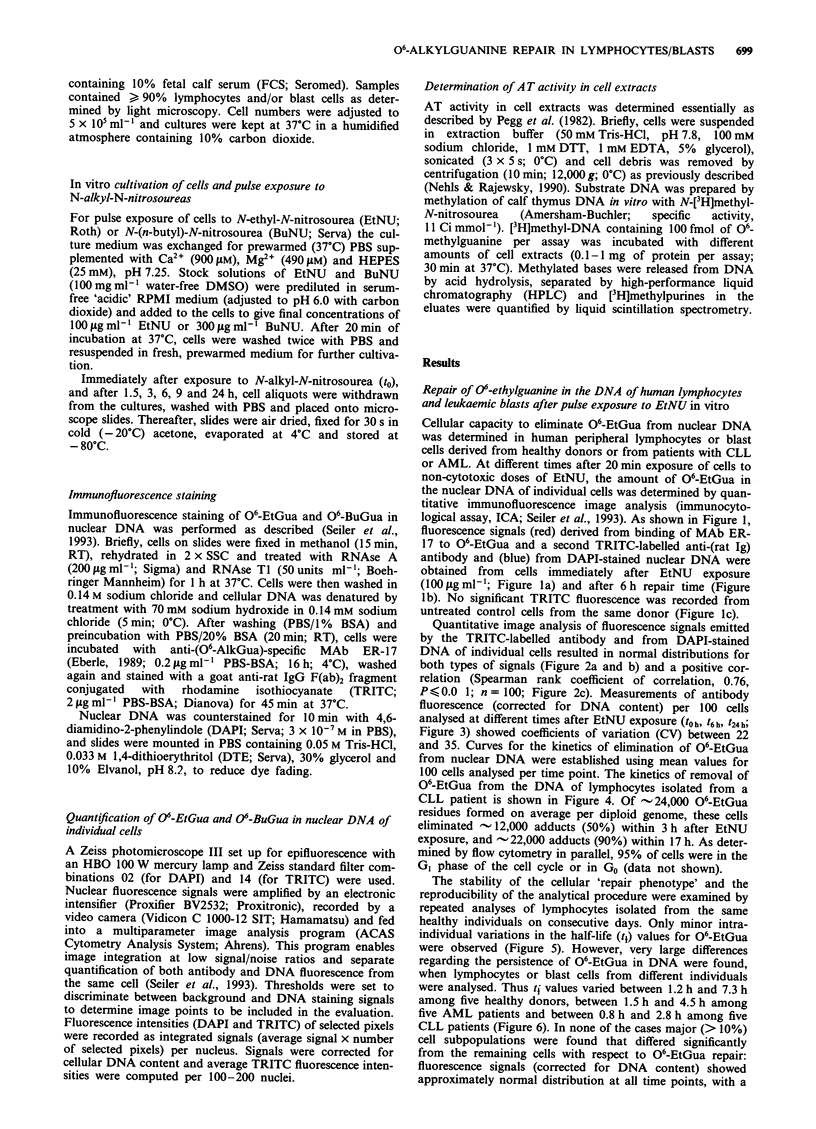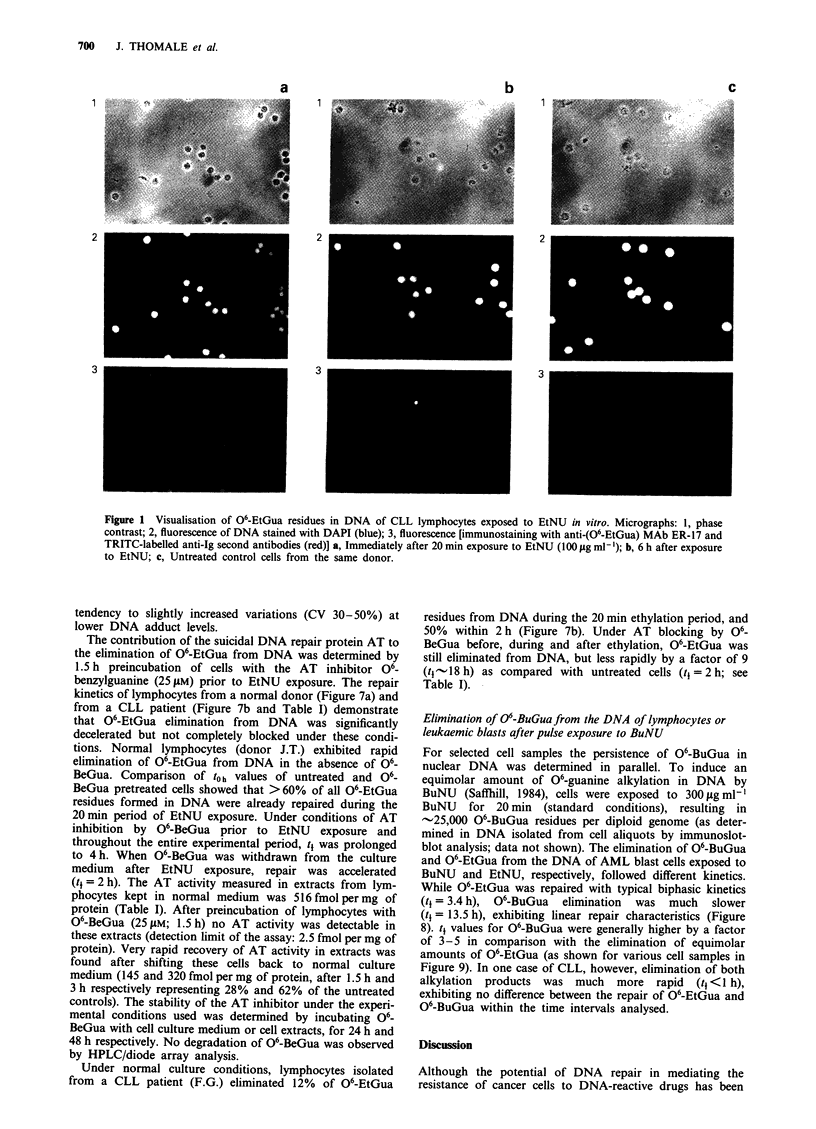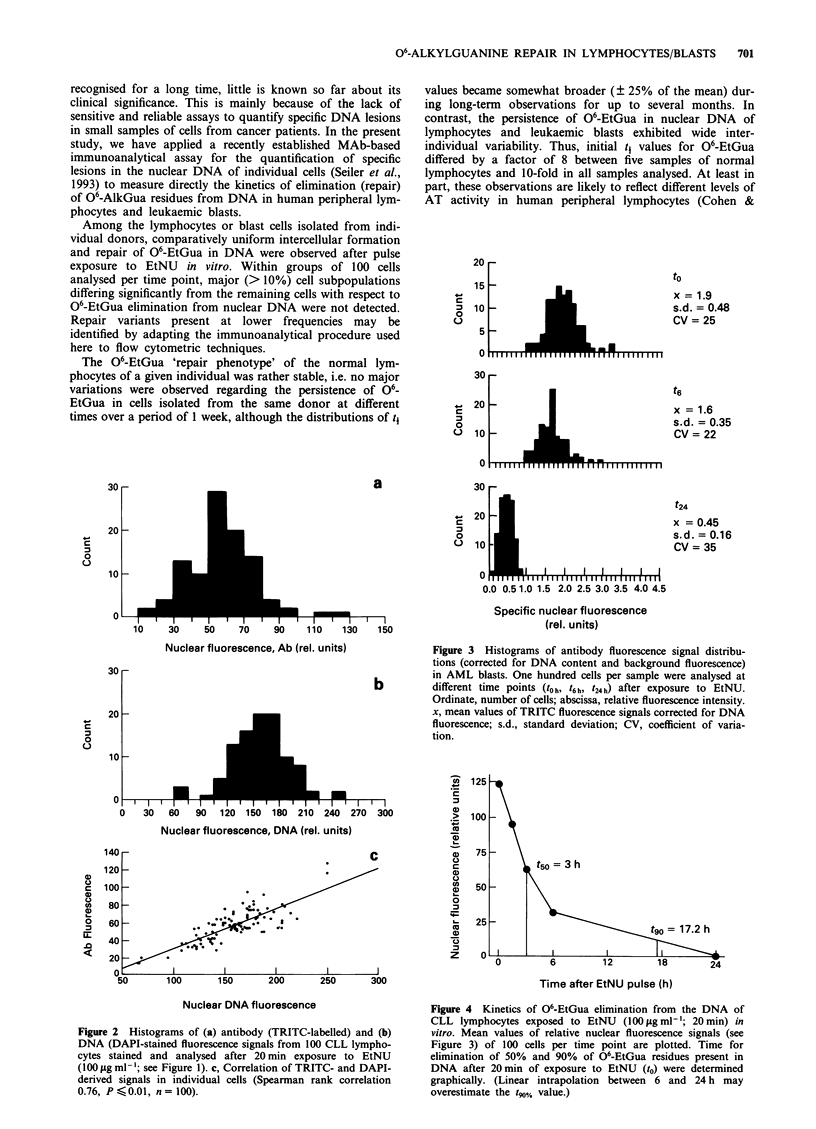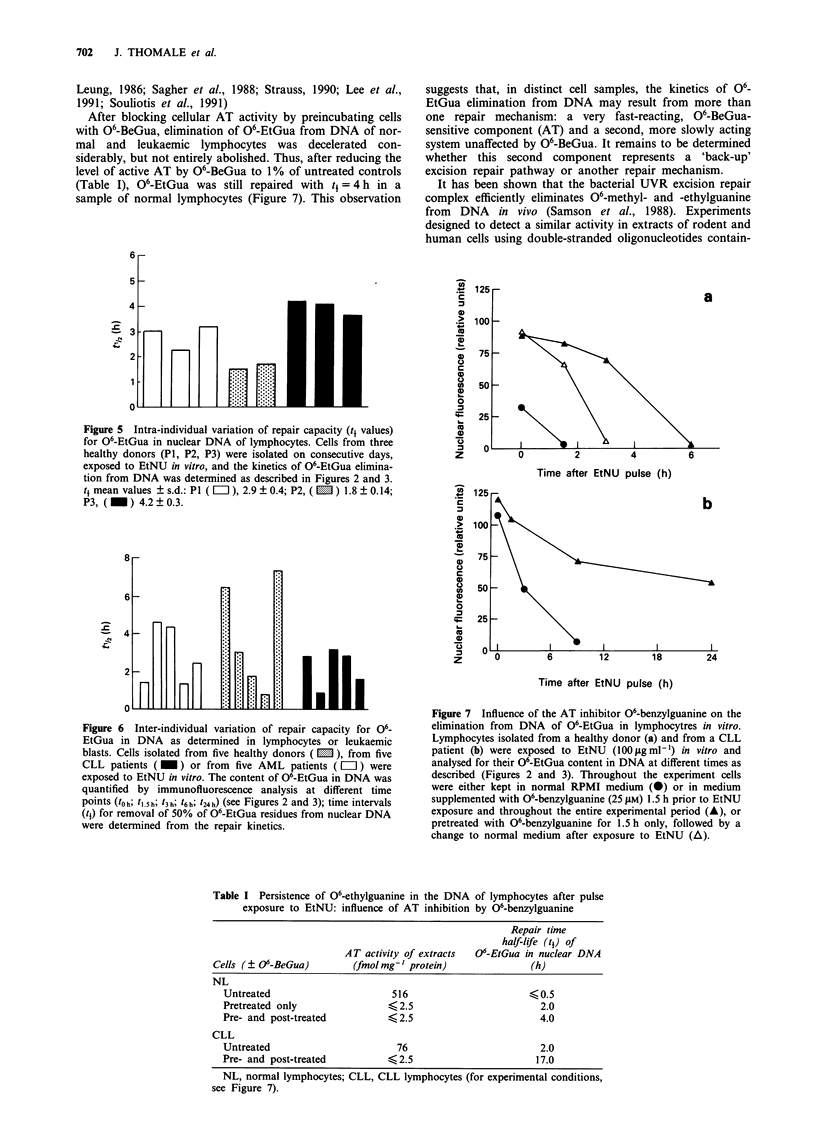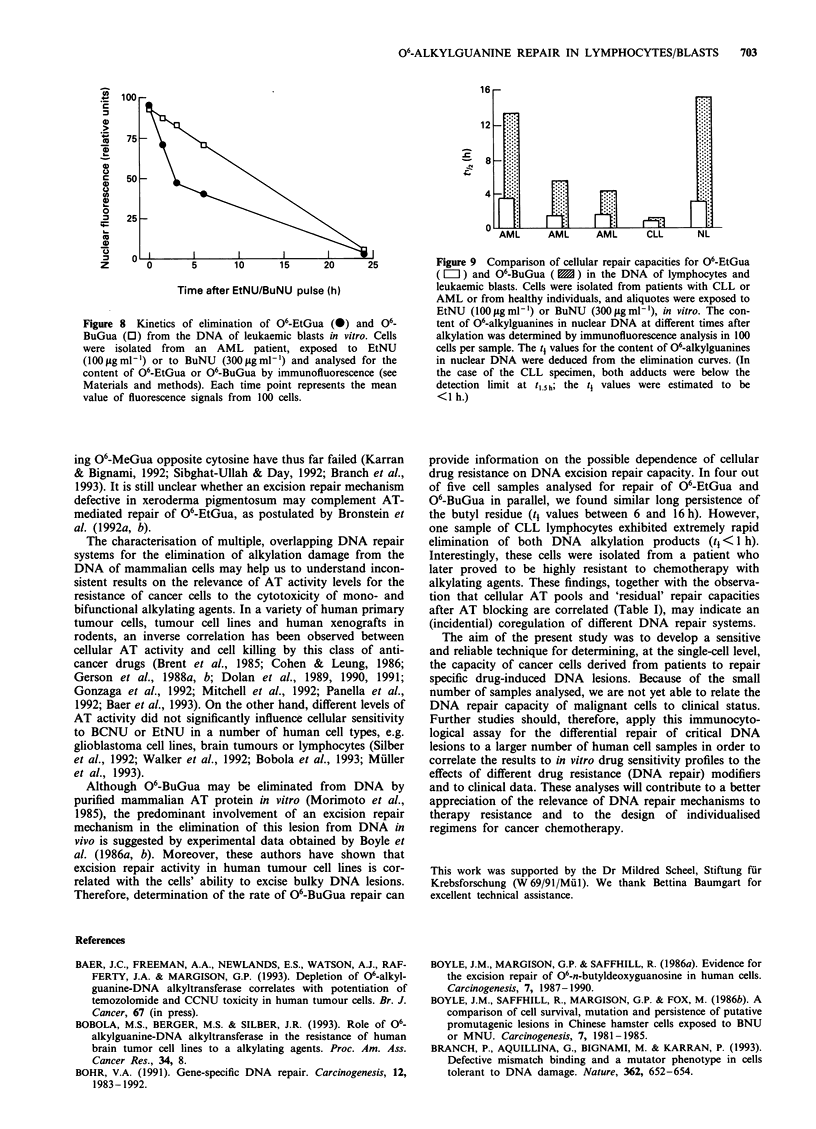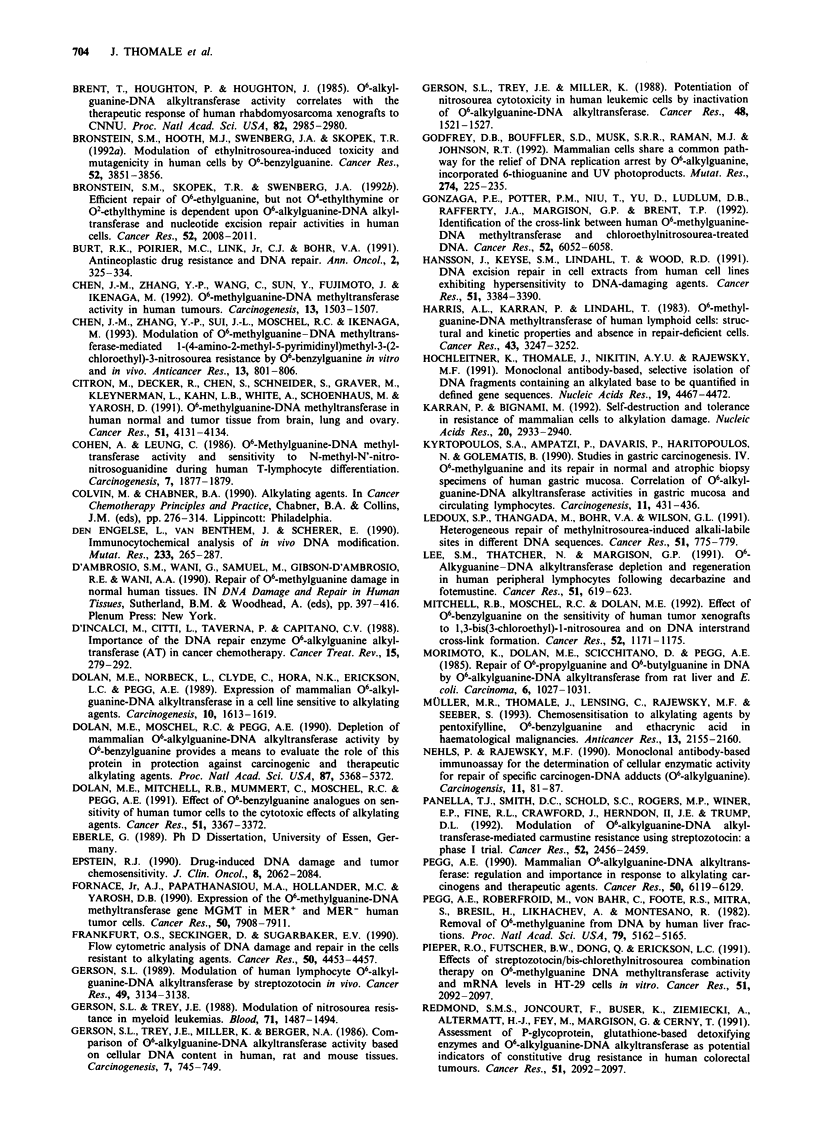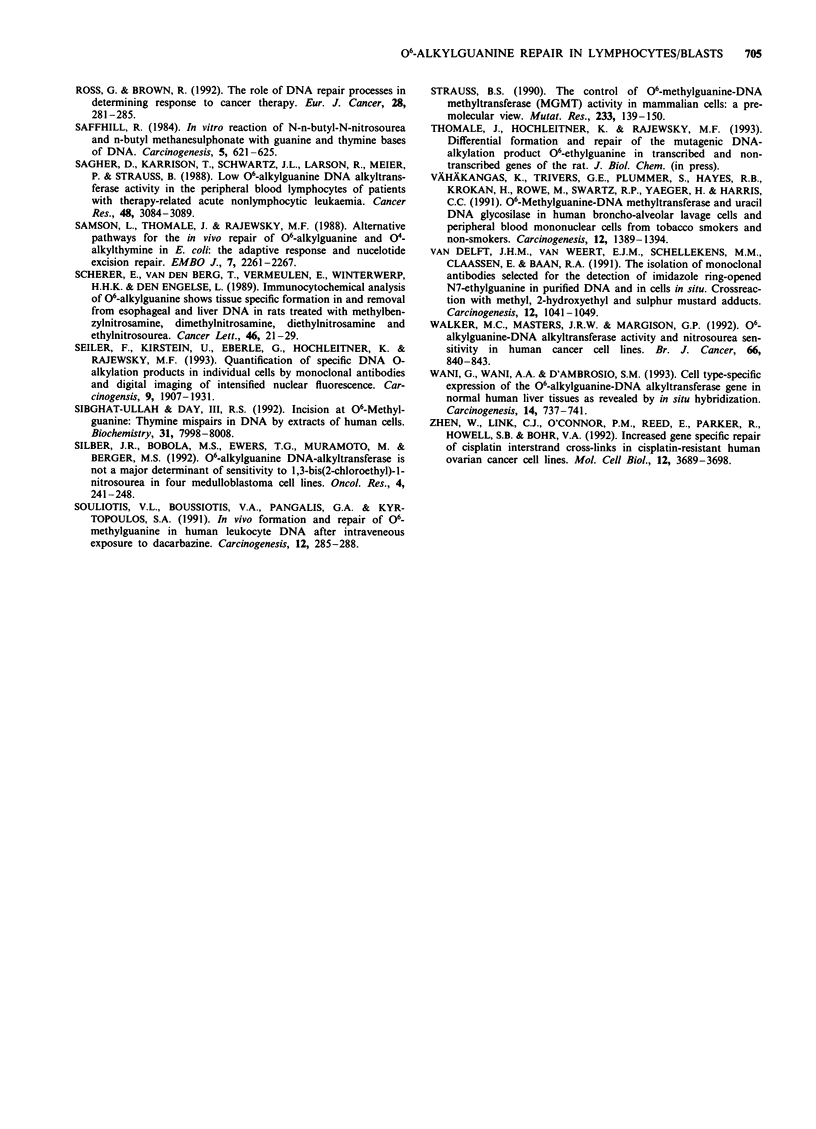# Repair of O6-alkylguanines in the nuclear DNA of human lymphocytes and leukaemic cells: analysis at the single-cell level.

**DOI:** 10.1038/bjc.1994.132

**Published:** 1994-04

**Authors:** J. Thomale, F. Seiler, M. R. Müller, S. Seeber, M. F. Rajewsky

**Affiliations:** Institute of Cell Biology (Cancer Research), West German Cancer Center Essen, University of Essen Medical School, Germany.

## Abstract

**Images:**


*Br. J. Cancer* (1994), **69**, 698–705
© Macmillan Press Ltd., 1994

### Repair of O$^6$-alkylguanines in the nuclear DNA of human lymphocytes and leukaemic cells: analysis at the single-cell level

J. Thomale[1], F. Seiler[1], M.R. Müller[2], S. Seeber[2] & M.F. Rajewsky[1]

[1]*Institute of Cell Biology (Cancer Research) and* [2]*Department of Medical Oncology, West German Cancer Center Essen, University of Essen Medical School, Hufelandstrasse 55, D-45122 Essen, Germany.*

**Summary** Inter-individual and cell–cell variability of repair of O$^6$-alkylguanines (O$^6$-AlkGua) in nuclear DNA was studied at the single-cell level in peripheral lymphocytes from healthy donors and in leukaemic cells isolated from patients with chronic lymphatic leukaemia (CLL) or acute myeloid leukaemia (AML). Cells were pulse exposed to *N*-ethyl- or *N*-(*n*-)butyl-*N*-nitrosourea *in vitro*, and O$^6$-AlkGua residues in DNA were quantified using an anti-(O$^6$-AlkGua) monoclonal antibody and electronically intensified fluorescence. The kinetics of O$^6$-AlkGua elimination revealed considerable inter-individual differences in O$^6$-ethylguanine (O$^6$-EtGua) half-life ($t_{½}$) values in DNA, ranging from 1.5 to 4.5 h (five AML patients), from 0.8 to 2.8 h (five CLL patients) and from 1.2 to 7.3 h (five healthy donors). The elimination from DNA of equimolar amounts of O$^6$-butylguanine was generally 3–5 times slower in comparison with O$^6$-EtGua. The $t_{½}$ values of individual samples varied in parallel for both DNA alkylation products. Upon preincubation with O$^6$-benzylguanine, the activity of the DNA repair protein O$^6$-alkylguanine–DNA alkyltransferase (AT) in both lymphocytes and leukaemic blasts was reduced to $\leqslant 1\%$. However, while the rate of O$^6$-EtGua elimination from DNA was decelerated it was not abolished, suggesting the possible involvement of additional repair systems that might be co-regulated with AT. Within individual samples, no major cell subpopulations were observed whose repair kinetics would differ significantly from the remaining cells.

Resistance to chemotherapeutic drugs and radiation represents a major obstacle in human cancer therapy. Since DNA is the most important target of many cytotoxic agents, the capacity for repair of specific, drug-induced DNA lesions may be an important determinant in the response of cancer cells to treatment, in addition to other mechanisms, such as drug transport and detoxification (reviewed, for example, by, Epstein, 1990; Burt *et al.*, 1991; Ross & Brown, 1992). However, comparatively little is known about the DNA repair capacity of malignant cells derived from patients. This is mainly because of the lack of sufficiently sensitive methods of quantifying specific DNA adducts and the rate of their elimination from DNA in small cell samples or in individual cells.

Mono- or bifunctional alkylating agents such as nitrosoureas, procarbazine, cyclophosphamide, mitomycin C, BCNU, busulphan and chlorambucil form cytotoxic and mutagenic DNA adducts via covalent bonds to nucleophilic sites, preferentially at the N$^7$- and O$^6$-atoms of guanine (Colvin & Chabner, 1990). Guanine-O$^6$ alkylation products in DNA are very efficiently repaired by a specific DNA alkyltransferase (AT; EC 2.1.1.63; Pegg, 1990) and, more slowly, by alternative pathways such as base or nucleotide excision (Boyle *et al.*, 1986*a*, *b*; Bronstein *et al.*, 1992*a*, *b*; Sibghat-Ullah & Day, 1992).

In human cells, e.g. normal cells or cells derived from tumours, autopsy material, surgical biopsies or fetal tissues, significant differences in cellular AT protein levels among different tissues have been reported (Gerson *et al.*, 1986; D'Incalci *et al.*, 1988; Fornace *et al.*, 1990; Vähäkangas *et al.*, 1991; Chen *et al.*, 1992; Wani *et al.*, 1993). Moreover, considerable inter-individual variability has been observed for a given type of cells (Kyrtopoulos *et al.*, 1990; Strauss, 1990; Citron *et al.*, 1991; Redmond *et al.*, 1991). Thus, in peripheral human lymphocytes inter-patient variations in AT levels up to a factor of 9 have been reported (Sagher *et al.*, 1988; Gerson, 1989; Lee *et al.*, 1991; Panella *et al.*, 1992). Plausible relationships have been proposed between cell type and individual sensitivity to the cytotoxic effects of alkylating or chloroalkylating agents on the one hand and the levels of cellular AT activity on the other (Brent *et al.*, 1985; Gerson *et al.*, 1988; Gerson & Trey, 1988; Dolan *et al.*, 1989; Pieper *et al.*, 1991; Panella *et al.*, 1992). Other experimental data indicate that cellular AT activity may not always be the most critical determinant of cellular resistance to alkylating agents such as CNUs or EtNU (Silber *et al.*, 1992; Bobola *et al.*, 1993; Chen *et al.*, 1993). Godfrey *et al.* (1992) have suggested that cellular resistance to the cytotoxic effect of O$^6$-alkylguanines (O$^6$-AlkGua) persisting in DNA could also be caused by mechanisms other than DNA repair, such as 'post-replication recovery'.

An increasing body of evidence indicates that DNA damage and the repair of specific DNA lesions is heterogeneous among individual cells (e.g. in biopsy material, Scherer *et al.*, 1989; Wani *et al.*, 1993) and throughout genomic DNA (Bohr, 1991; Le Doux *et al.*, 1991; Thomale *et al.*, 1993). However, only very recently, sufficiently sensitive analytical methods have become available which permit us to quantify specific drug-induced DNA lesions in defined gene sequences (Hochleitner *et al.*, 1991; Zhen *et al.*, 1992) and in single cells (Frankfurt *et al.*, 1990; Van Delft *et al.*, 1991; Seiler *et al.*, 1993). In the present study, we have applied a newly developed, monoclonal antibody (MAb)-based immunocytological assay (ICA; Seiler *et al.*, 1993) to measure the repair kinetics of O$^6$-AlkGua in the nuclear DNA of individual human lymphocytes and leukaemic blasts after pulse exposure to *N*-alkyl-*N*-nitrosoureas. This class of compounds is particularly suitable as prototype substances because the reaction kinetics and all major reaction products with DNA are well characterised. Moreover, we have determined the influence of O$^6$-benzylguanine (O$^6$-BeGua), an inhibitor of cellular AT activity (Dolan *et al.*, 1990), on the persistence of O$^6$-alkylguanines in the DNA of these cells.

### Materials and methods

### *Isolation of peripheral lymphocytes or leukaemic blasts*

Heparinised blood (10 ml) obtained from patients with AML or CLL before chemotherapy or from healthy donors was layered onto 10 ml of Ficoll–Hypaque and centrifuged for 25 min at 200 *g* at room temperature (RT). Cells at the interphase were removed, washed twice in phosphate-buffered saline (PBS) and resuspended in RPMI-1640 medium (Gibco)

Correspondence: J. Thomale.
Received 27 September 1993; and in revised form 26 November 1993.

containing 10% fetal calf serum (FCS; Seromed). Samples contained $\geq 90\%$ lymphocytes and/or blast cells as determined by light microscopy. Cell numbers were adjusted to $5 \times 10^5$ ml$^{-1}$ and cultures were kept at 37°C in a humidified atmosphere containing 10% carbon dioxide.

### *In vitro* cultivation of cells and pulse exposure to N-alkyl-N-nitrosoureas

For pulse exposure of cells to *N*-ethyl-*N*-nitrosourea (EtNU; Roth) or *N*-(*n*-butyl)-*N*-nitrosourea (BuNU; Serva) the culture medium was exchanged for prewarmed (37°C) PBS supplemented with $Ca^{2+}$ (900 μM), $Mg^{2+}$ (490 μM) and HEPES (25 mM), pH 7.25. Stock solutions of EtNU and BuNU (100 mg ml$^{-1}$ water-free DMSO) were prediluted in serum-free 'acidic' RPMI medium (adjusted to pH 6.0 with carbon dioxide) and added to the cells to give final concentrations of 100 μg ml$^{-1}$ EtNU or 300 μg ml$^{-1}$ BuNU. After 20 min of incubation at 37°C, cells were washed twice with PBS and resuspended in fresh, prewarmed medium for further cultivation.

Immediately after exposure to *N*-alkyl-*N*-nitrosourea ($t_0$), and after 1.5, 3, 6, 9 and 24 h, cell aliquots were withdrawn from the cultures, washed with PBS and placed onto microscope slides. Thereafter, slides were air dried, fixed for 30 s in cold (−20°C) acetone, evaporated at 4°C and stored at −80°C.

### *Immunofluorescence staining*

Immunofluorescence staining of $O^6$-EtGua and $O^6$-BuGua in nuclear DNA was performed as described (Seiler *et al.*, 1993). Briefly, cells on slides were fixed in methanol (15 min, RT), rehydrated in 2 × SSC and treated with RNAse A (200 μg ml$^{-1}$; Sigma) and RNAse T1 (50 units ml$^{-1}$; Boehringer Mannheim) for 1 h at 37°C. Cells were then washed in 0.14 M sodium chloride and cellular DNA was denatured by treatment with 70 mM sodium hydroxide in 0.14 mM sodium chloride (5 min; 0°C). After washing (PBS/1% BSA) and preincubation with PBS/20% BSA (20 min; RT), cells were incubated with anti-($O^6$-AlkGua)-specific MAb ER-17 (Eberle, 1989; 0.2 μg ml$^{-1}$ PBS-BSA; 16 h; 4°C), washed again and stained with a goat anti-rat IgG F(ab)$_2$ fragment conjugated with rhodamine isothiocyanate (TRITC; 2 μg ml$^{-1}$ PBS-BSA; Dianova) for 45 min at 37°C.

Nuclear DNA was counterstained for 10 min with 4,6-diamidino-2-phenylindole (DAPI; Serva; $3 \times 10^{-7}$ M in PBS), and slides were mounted in PBS containing 0.05 M Tris-HCl, 0.033 M 1,4-dithioerythritol (DTE; Serva), 30% glycerol and 10% Elvanol, pH 8.2, to reduce dye fading.

### *Quantification of $O^6$-EtGua and $O^6$-BuGua in nuclear DNA of individual cells*

A Zeiss photomicroscope III set up for epifluorescence with an HBO 100 W mercury lamp and Zeiss standard filter combinations 02 (for DAPI) and 14 (for TRITC) were used. Nuclear fluorescence signals were amplified by an electronic intensifier (Proxifier BV2532; Proxitronic), recorded by a video camera (Vidicon C 1000-12 SIT; Hamamatsu) and fed into a multiparameter image analysis program (ACAS Cytometry Analysis System; Ahrens). This program enables image integration at low signal/noise ratios and separate quantification of both antibody and DNA fluorescence from the same cell (Seiler *et al.*, 1993). Thresholds were set to discriminate between background and DNA staining signals to determine image points to be included in the evaluation. Fluorescence intensities (DAPI and TRITC) of selected pixels were recorded as integrated signals (average signal × number of selected pixels) per nucleus. Signals were corrected for cellular DNA content and average TRITC fluorescence intensities were computed per 100–200 nuclei.

### *Determination of AT activity in cell extracts*

AT activity in cell extracts was determined essentially as described by Pegg *et al.* (1982). Briefly, cells were suspended in extraction buffer (50 mM Tris-HCl, pH 7.8, 100 mM sodium chloride, 1 mM DTT, 1 mM EDTA, 5% glycerol), sonicated (3 × 5 s; 0°C) and cell debris was removed by centrifugation (10 min; 12,000 *g*; 0°C) as previously described (Nehls & Rajewsky, 1990). Substrate DNA was prepared by methylation of calf thymus DNA *in vitro* with *N*-[$^3$H]methyl-*N*-nitrosourea (Amersham-Buchler; specific activity, 11 Ci mmol$^{-1}$). [$^3$H]methyl-DNA containing 100 fmol of $O^6$-methylguanine per assay was incubated with different amounts of cell extracts (0.1–1 mg of protein per assay; 30 min at 37°C). Methylated bases were released from DNA by acid hydrolysis, separated by high-performance liquid chromatography (HPLC) and [$^3$H]methylpurines in the eluates were quantified by liquid scintillation spectrometry.

### Results

### *Repair of $O^6$-ethylguanine in the DNA of human lymphocytes and leukaemic blasts after pulse exposure to EtNU* in vitro

Cellular capacity to eliminate $O^6$-EtGua from nuclear DNA was determined in human peripheral lymphocytes or blast cells derived from healthy donors or from patients with CLL or AML. At different times after 20 min exposure of cells to non-cytotoxic doses of EtNU, the amount of $O^6$-EtGua in the nuclear DNA of individual cells was determined by quantitative immunofluorescence image analysis (immunocytological assay, ICA; Seiler *et al.*, 1993). As shown in Figure 1, fluorescence signals (red) derived from binding of MAb ER-17 to $O^6$-EtGua and a second TRITC-labelled anti-(rat Ig) antibody and (blue) from DAPI-stained nuclear DNA were obtained from cells immediately after EtNU exposure (100 μg ml$^{-1}$; Figure 1a) and after 6 h repair time (Figure 1b). No significant TRITC fluorescence was recorded from untreated control cells from the same donor (Figure 1c).

Quantitative image analysis of fluorescence signals emitted by the TRITC-labelled antibody and from DAPI-stained DNA of individual cells resulted in normal distributions for both types of signals (Figure 2a and b) and a positive correlation (Spearman rank coefficient of correlation, 0.76, $P \leq 0.0$ 1; $n = 100$; Figure 2c). Measurements of antibody fluorescence (corrected for DNA content) per 100 cells analysed at different times after EtNU exposure ($t_{0\,h}$, $t_{6\,h}$, $t_{24\,h}$; Figure 3) showed coefficients of variation (CV) between 22 and 35. Curves for the kinetics of elimination of $O^6$-EtGua from nuclear DNA were established using mean values for 100 cells analysed per time point. The kinetics of removal of $O^6$-EtGua from the DNA of lymphocytes isolated from a CLL patient is shown in Figure 4. Of ~24,000 $O^6$-EtGua residues formed on average per diploid genome, these cells eliminated ~12,000 adducts (50%) within 3 h after EtNU exposure, and ~22,000 adducts (90%) within 17 h. As determined by flow cytometry in parallel, 95% of cells were in the $G_1$ phase of the cell cycle or in $G_0$ (data not shown).

The stability of the cellular 'repair phenotype' and the reproducibility of the analytical procedure were examined by repeated analyses of lymphocytes isolated from the same healthy individuals on consecutive days. Only minor intra-individual variations in the half-life ($t_{\frac{1}{2}}$) values for $O^6$-EtGua were observed (Figure 5). However, very large differences regarding the persistence of $O^6$-EtGua in DNA were found, when lymphocytes or blast cells from different individuals were analysed. Thus $t_{\frac{1}{2}}$ values varied between 1.2 h and 7.3 h among five healthy donors, between 1.5 h and 4.5 h among five AML patients and between 0.8 h and 2.8 h among five CLL patients (Figure 6). In none of the cases major (> 10%) cell subpopulations were found that differed significantly from the remaining cells with respect to $O^6$-EtGua repair: fluorescence signals (corrected for DNA content) showed approximately normal distribution at all time points, with a

**Figure 1** Visualisation of $O^6$-EtGua residues in DNA of CLL lymphocytes exposed to EtNU *in vitro*. Micrographs: 1, phase contrast; 2, fluorescence of DNA stained with DAPI (blue); 3, fluorescence [immunostaining with anti-($O^6$-EtGua) MAb ER-17 and TRITC-labelled anti-Ig second antibodies (red)] **a**, Immediately after 20 min exposure to EtNU (100 $\mu$g ml$^{-1}$); **b**, 6 h after exposure to EtNU; **c**, Untreated control cells from the same donor.

tendency to slightly increased variations (CV 30–50%) at lower DNA adduct levels.

The contribution of the suicidal DNA repair protein AT to the elimination of $O^6$-EtGua from DNA was determined by 1.5 h preincubation of cells with the AT inhibitor $O^6$-benzylguanine (25 $\mu$M) prior to EtNU exposure. The repair kinetics of lymphocytes from a normal donor (Figure 7a) and from a CLL patient (Figure 7b and Table I) demonstrate that $O^6$-EtGua elimination from DNA was significantly decelerated but not completely blocked under these conditions. Normal lymphocytes (donor J.T.) exhibited rapid elimination of $O^6$-EtGua from DNA in the absence of $O^6$-BeGua. Comparison of $t_{0\,h}$ values of untreated and $O^6$-BeGua pretreated cells showed that >60% of all $O^6$-EtGua residues formed in DNA were already repaired during the 20 min period of EtNU exposure. Under conditions of AT inhibition by $O^6$-BeGua prior to EtNU exposure and throughout the entire experimental period, $t_{½}$ was prolonged to 4 h. When $O^6$-BeGua was withdrawn from the culture medium after EtNU exposure, repair was accelerated ($t_{½} = 2$ h). The AT activity measured in extracts from lymphocytes kept in normal medium was 516 fmol per mg of protein (Table I). After preincubation of lymphocytes with $O^6$-BeGua (25 $\mu$M; 1.5 h) no AT activity was detectable in these extracts (detection limit of the assay: 2.5 fmol per mg of protein). Very rapid recovery of AT activity in extracts was found after shifting these cells back to normal culture medium (145 and 320 fmol per mg of protein, after 1.5 h and 3 h respectively representing 28% and 62% of the untreated controls). The stability of the AT inhibitor under the experimental conditions used was determined by incubating $O^6$-BeGua with cell culture medium or cell extracts, for 24 h and 48 h respectively. No degradation of $O^6$-BeGua was observed by HPLC/diode array analysis.

Under normal culture conditions, lymphocytes isolated from a CLL patient (F.G.) eliminated 12% of $O^6$-EtGua residues from DNA during the 20 min ethylation period, and 50% within 2 h (Figure 7b). Under AT blocking by $O^6$-BeGua before, during and after ethylation, $O^6$-EtGua was still eliminated from DNA, but less rapidly by a factor of 9 ($t_{½} \sim 18$ h) as compared with untreated cells ($t_{½} = 2$ h; see Table I).

### *Elimination of $O^6$-BuGua from the DNA of lymphocytes or leukaemic blasts after pulse exposure to BuNU*

For selected cell samples the persistence of $O^6$-BuGua in nuclear DNA was determined in parallel. To induce an equimolar amount of $O^6$-guanine alkylation in DNA by BuNU (Saffhill, 1984), cells were exposed to 300 $\mu$g ml$^{-1}$ BuNU for 20 min (standard conditions), resulting in ~25,000 $O^6$-BuGua residues per diploid genome (as determined in DNA isolated from cell aliquots by immunoslot-blot analysis; data not shown). The elimination of $O^6$-BuGua and $O^6$-EtGua from the DNA of AML blast cells exposed to BuNU and EtNU, respectively, followed different kinetics. While $O^6$-EtGua was repaired with typical biphasic kinetics ($t_{½} = 3.4$ h), $O^6$-BuGua elimination was much slower ($t_{½} = 13.5$ h), exhibiting linear repair characteristics (Figure 8). $t_{½}$ values for $O^6$-BuGua were generally higher by a factor of 3–5 in comparison with the elimination of equimolar amounts of $O^6$-EtGua (as shown for various cell samples in Figure 9). In one case of CLL, however, elimination of both alkylation products was much more rapid ($t_{½} < 1$ h), exhibiting no difference between the repair of $O^6$-EtGua and $O^6$-BuGua within the time intervals analysed.

### Discussion

Although the potential of DNA repair in mediating the resistance of cancer cells to DNA-reactive drugs has been recognised for a long time, little is known so far about its clinical significance. This is mainly because of the lack of sensitive and reliable assays to quantify specific DNA lesions in small samples of cells from cancer patients. In the present study, we have applied a recently established MAb-based immunoanalytical assay for the quantification of specific lesions in the nuclear DNA of individual cells (Seiler *et al.*, 1993) to measure directly the kinetics of elimination (repair) of O$^6$-AlkGua residues from DNA in human peripheral lymphocytes and leukaemic blasts.

Among the lymphocytes or blast cells isolated from individual donors, comparatively uniform intercellular formation and repair of O$^6$-EtGua in DNA were observed after pulse exposure to EtNU *in vitro*. Within groups of 100 cells analysed per time point, major (> 10%) cell subpopulations differing significantly from the remaining cells with respect to O$^6$-EtGua elimination from nuclear DNA were not detected. Repair variants present at lower frequencies may be identified by adapting the immunoanalytical procedure used here to flow cytometric techniques.

The O$^6$-EtGua 'repair phenotype' of the normal lymphocytes of a given individual was rather stable, i.e. no major variations were observed regarding the persistence of O$^6$-EtGua in cells isolated from the same donor at different times over a period of 1 week, although the distributions of $t_{½}$ values became somewhat broader (± 25% of the mean) during long-term observations for up to several months. In contrast, the persistence of O$^6$-EtGua in nuclear DNA of lymphocytes and leukaemic blasts exhibited wide inter-individual variability. Thus, initial $t_{½}$ values for O$^6$-EtGua differed by a factor of 8 between five samples of normal lymphocytes and 10-fold in all samples analysed. At least in part, these observations are likely to reflect different levels of AT activity in human peripheral lymphocytes (Cohen &

**Figure 2** Histograms of (**a**) antibody (TRITC-labelled) and (**b**) DNA (DAPI-stained fluorescence signals from 100 CLL lymphocytes stained and analysed after 20 min exposure to EtNU (100 μg ml$^{-1}$; see Figure 1). **c**, Correlation of TRITC- and DAPI-derived signals in individual cells (Spearman rank correlation 0.76, $P \leqslant 0.01$, $n = 100$).

**Figure 3** Histograms of antibody fluorescence signal distributions (corrected for DNA content and background fluorescence) in AML blasts. One hundred cells per sample were analysed at different time points ($t_{0\,h}$, $t_{6\,h}$, $t_{24\,h}$) after exposure to EtNU. Ordinate, number of cells; abscissa, relative fluorescence intensity. $x$, mean values of TRITC fluorescence signals corrected for DNA fluorescence; s.d., standard deviation; CV, coefficient of variation.

**Figure 4** Kinetics of O$^6$-EtGua elimination from the DNA of CLL lymphocytes exposed to EtNU (100 μg ml$^{-1}$; 20 min) *in vitro*. Mean values of relative nuclear fluorescence signals (see Figure 3) of 100 cells per time point are plotted. Time for elimination of 50% and 90% of O$^6$-EtGua residues present in DNA after 20 min of exposure to EtNU ($t_0$) were determined graphically. (Linear intrapolation between 6 and 24 h may overestimate the $t_{90\%}$ value.)

Leung, 1986; Sagher et al., 1988; Strauss, 1990; Lee et al., 1991; Souliotis et al., 1991)

After blocking cellular AT activity by preincubating cells with O[6]-BeGua, elimination of O[6]-EtGua from DNA of normal and leukaemic lymphocytes was decelerated considerably, but not entirely abolished. Thus, after reducing the level of active AT by O[6]-BeGua to 1% of untreated controls (Table I), O[6]-EtGua was still repaired with $t_{\frac{1}{2}} = 4$ h in a sample of normal lymphocytes (Figure 7). This observation suggests that, in distinct cell samples, the kinetics of O[6]-EtGua elimination from DNA may result from more than one repair mechanism: a very fast-reacting, O[6]-BeGua-sensitive component (AT) and a second, more slowly acting system unaffected by O[6]-BeGua. It remains to be determined whether this second component represents a 'back-up' excision repair pathway or another repair mechanism.

It has been shown that the bacterial UVR excision repair complex efficiently eliminates O[6]-methyl- and -ethylguanine from DNA in vivo (Samson et al., 1988). Experiments designed to detect a similar activity in extracts of rodent and human cells using double-stranded oligonucleotides contain-

**Figure 5** Intra-individual variation of repair capacity ($t_{\frac{1}{2}}$ values) for O[6]-EtGua in nuclear DNA of lymphocytes. Cells from three healthy donors (P1, P2, P3) were isolated on consecutive days, exposed to EtNU *in vitro*, and the kinetics of O[6]-EtGua elimination from DNA was determined as described in Figures 2 and 3. $t_{\frac{1}{2}}$ mean values ± s.d.: P1 ( □ ), 2.9 ± 0.4; P2, ( ▒ ) 1.8 ± 0.14; P3, ( ■ ) 4.2 ± 0.3.

**Figure 6** Inter-individual variation of repair capacity for O[6]-EtGua in DNA as determined in lymphocytes or leukaemic blasts. Cells isolated from five healthy donors ( ▒ ), from five CLL patients ( ■ ) or from five AML patients ( □ ) were exposed to EtNU *in vitro*. The content of O[6]-EtGua in DNA was quantified by immunofluorescence analysis at different time points ($t_{0\,h}$; $t_{1.5\,h}$; $t_{3\,h}$; $t_{6\,h}$; $t_{24\,h}$) (see Figures 2 and 3); time intervals ($t_{\frac{1}{2}}$) for removal of 50% of O[6]-EtGua residues from nuclear DNA were determined from the repair kinetics.

**Figure 7** Influence of the AT inhibitor O[6]-benzylguanine on the elimination from DNA of O[6]-EtGua in lymphocytres *in vitro*. Lymphocytes isolated from a healthy donor (**a**) and from a CLL patient (**b**) were exposed to EtNU (100 μg ml$^{-1}$) *in vitro* and analysed for their O[6]-EtGua content in DNA at different times as described (Figures 2 and 3). Throughout the experiment cells were either kept in normal RPMI medium (●) or in medium supplemented with O[6]-benzylguanine (25 μM) 1.5 h prior to EtNU exposure and throughout the entire experimental period (▲), or pretreated with O[6]-benzylguanine for 1.5 h only, followed by a change to normal medium after exposure to EtNU (△).

**Table I** Persistence of O[6]-ethylguanine in the DNA of lymphocytes after pulse exposure to EtNU: influence of AT inhibition by O[6]-benzylguanine

| *Cells (± O[6]-BeGua)* | *AT activity of extracts (fmol mg$^{-1}$ protein)* | *Repair time half-life ($t_{\frac{1}{2}}$) of O[6]-EtGua in nuclear DNA (h)* |
|---|---|---|
| NL | | |
| Untreated | 516 | ⩽0.5 |
| Pretreated only | ⩽2.5 | 2.0 |
| Pre- and post-treated | ⩽2.5 | 4.0 |
| CLL | | |
| Untreated | 76 | 2.0 |
| Pre- and post-treated | ⩽2.5 | 17.0 |

NL, normal lymphocytes; CLL, CLL lymphocytes (for experimental conditions, see Figure 7).

**Figure 8** Kinetics of elimination of $O^6$-EtGua (●) and $O^6$-BuGua (□) from the DNA of leukaemic blasts *in vitro*. Cells were isolated from an AML patient, exposed to EtNU ($100\,\mu g\,ml^{-1}$) or to BuNU ($300\,\mu g\,ml^{-1}$) and analysed for the content of $O^6$-EtGua or $O^6$-BuGua by immunofluorescence (see Materials and methods). Each time point represents the mean value of fluorescence signals from 100 cells.

**Figure 9** Comparison of cellular repair capacities for $O^6$-EtGua (□) and $O^6$-BuGua (▨) in the DNA of lymphocytes and leukaemic blasts. Cells were isolated from patients with CLL or AML or from healthy individuals, and aliquotes were exposed to EtNU ($100\,\mu g\,ml^{-1}$) or BuNU ($300\,\mu g\,ml^{-1}$), *in vitro*. The content of $O^6$-alkylguanines in nuclear DNA at different times after alkylation was determined by immunofluorescence analysis in 100 cells per sample. The $t_{½}$ values for the content of $O^6$-alkylguanines in nuclear DNA were deduced from the elimination curves. (In the case of the CLL specimen, both adducts were below the detection limit at $t_{1.5\,h}$; the $t_{½}$ values were estimated to be $<1$ h.)

ing $O^6$-MeGua opposite cytosine have thus far failed (Karran & Bignami, 1992; Sibghat-Ullah & Day, 1992; Branch *et al.*, 1993). It is still unclear whether an excision repair mechanism defective in xeroderma pigmentosum may complement AT-mediated repair of $O^6$-EtGua, as postulated by Bronstein *et al.* (1992*a*, *b*).

The characterisation of multiple, overlapping DNA repair systems for the elimination of alkylation damage from the DNA of mammalian cells may help us to understand inconsistent results on the relevance of AT activity levels for the resistance of cancer cells to the cytotoxicity of mono- and bifunctional alkylating agents. In a variety of human primary tumour cells, tumour cell lines and human xenografts in rodents, an inverse correlation has been observed between cellular AT activity and cell killing by this class of anticancer drugs (Brent *et al.*, 1985; Cohen & Leung, 1986; Gerson *et al.*, 1988*a*, *b*; Dolan *et al.*, 1989, 1990, 1991; Gonzaga *et al.*, 1992; Mitchell *et al.*, 1992; Panella *et al.*, 1992; Baer *et al.*, 1993). On the other hand, different levels of AT activity did not significantly influence cellular sensitivity to BCNU or EtNU in a number of human cell types, e.g. glioblastoma cell lines, brain tumours or lymphocytes (Silber *et al.*, 1992; Walker *et al.*, 1992; Bobola *et al.*, 1993; Müller *et al.*, 1993).

Although $O^6$-BuGua may be eliminated from DNA by purified mammalian AT protein *in vitro* (Morimoto *et al.*, 1985), the predominant involvement of an excision repair mechanism in the elimination of this lesion from DNA *in vivo* is suggested by experimental data obtained by Boyle *et al.* (1986*a*, *b*). Moreover, these authors have shown that excision repair activity in human tumour cell lines is correlated with the cells' ability to excise bulky DNA lesions. Therefore, determination of the rate of $O^6$-BuGua repair can provide information on the possible dependence of cellular drug resistance on DNA excision repair capacity. In four out of five cell samples analysed for repair of $O^6$-EtGua and $O^6$-BuGua in parallel, we found similar long persistence of the butyl residue ($t_{½}$ values between 6 and 16 h). However, one sample of CLL lymphocytes exhibited extremely rapid elimination of both DNA alkylation products ($t_{½} < 1$ h). Interestingly, these cells were isolated from a patient who later proved to be highly resistant to chemotherapy with alkylating agents. These findings, together with the observation that cellular AT pools and 'residual' repair capacities after AT blocking are correlated (Table I), may indicate an (incidential) coregulation of different DNA repair systems.

The aim of the present study was to develop a sensitive and reliable technique for determining, at the single-cell level, the capacity of cancer cells derived from patients to repair specific drug-induced DNA lesions. Because of the small number of samples analysed, we are not yet able to relate the DNA repair capacity of malignant cells to clinical status. Further studies should, therefore, apply this immunocytological assay for the differential repair of critical DNA lesions to a larger number of human cell samples in order to correlate the results to *in vitro* drug sensitivity profiles to the effects of different drug resistance (DNA repair) modifiers and to clinical data. These analyses will contribute to a better appreciation of the relevance of DNA repair mechanisms to therapy resistance and to the design of individualised regimens for cancer chemotherapy.

This work was supported by the Dr Mildred Scheel, Stiftung für Krebsforschung (W 69/91/Mül). We thank Bettina Baumgart for excellent technical assistance.

### References

BAER, J.C., FREEMAN, A.A., NEWLANDS, E.S., WATSON, A.J., RAFFERTY, J.A. & MARGISON, G.P. (1993). Depletion of $O^6$-alkylguanine-DNA alkyltransferase correlates with potentiation of temozolomide and CCNU toxicity in human tumour cells. *Br. J. Cancer*, **67** (in press).

BOBOLA, M.S., BERGER, M.S. & SILBER, J.R. (1993). Role of $O^6$-alkylguanine-DNA alkyltransferase in the resistance of human brain tumor cell lines to a alkylating agents. *Proc. Am. Ass. Cancer Res.*, **34**, 8.

BOHR, V.A. (1991). Gene-specific DNA repair. *Carcinogenesis*, **12**, 1983-1992.

BOYLE, J.M., MARGISON, G.P. & SAFFHILL, R. (1986*a*). Evidence for the excision repair of $O^6$-*n*-butyldeoxyguanosine in human cells. *Carcinogenesis*, **7**, 1987-1990.

BOYLE, J.M., SAFFHILL, R., MARGISON, G.P. & FOX, M. (1986*b*). A comparison of cell survival, mutation and persistence of putative promutagenic lesions in Chinese hamster cells exposed to BNU or MNU. *Carcinogenesis*, **7**, 1981-1985.

BRANCH, P., AQUILLINA, G., BIGNAMI, M. & KARRAN, P. (1993). Defective mismatch binding and a mutator phenotype in cells tolerant to DNA damage. *Nature*, **362**, 652-654.

BRENT, T., HOUGHTON, P. & HOUGHTON, J. (1985). O$^6$-alkylguanine-DNA alkyltransferase activity correlates with the therapeutic response of human rhabdomyosarcoma xenografts to CNNU. *Proc. Natl Acad. Sci. USA*, **82**, 2985–2980.

BRONSTEIN, S.M., HOOTH, M.J., SWENBERG, J.A. & SKOPEK, T.R. (1992*a*). Modulation of ethylnitrosourea-induced toxicity and mutagenicity in human cells by O$^6$-benzylguanine. *Cancer Res.*, **52**, 3851–3856.

BRONSTEIN, S.M., SKOPEK, T.R. & SWENBERG, J.A. (1992*b*). Efficient repair of O$^6$-ethylguanine, but not O$^4$-ethylthymine or O$^2$-ethylthymine is dependent upon O$^6$-alkylguanine-DNA alkyltransferase and nucleotide excision repair activities in human cells. *Cancer Res.*, **52**, 2008–2011.

BURT, R.K., POIRIER, M.C., LINK, Jr, C.J. & BOHR, V.A. (1991). Antineoplastic drug resistance and DNA repair. *Ann. Oncol.*, **2**, 325–334.

CHEN, J.-M., ZHANG, Y.-P., WANG, C., SUN, Y., FUJIMOTO, J. & IKENAGA, M. (1992). O$^6$-methylguanine-DNA methyltransferase activity in human tumours. *Carcinogenesis*, **13**, 1503–1507.

CHEN, J.-M., ZHANG, Y.-P., SUI, J.-L., MOSCHEL, R.C. & IKENAGA, M. (1993). Modulation of O$^6$-methylguanine–DNA methyltransferase-mediated 1-(4-amino-2-methyl-5-pyrimidinyl)methyl-3-(2-chloroethyl)-3-nitrosourea resistance by O$^6$-benzylguanine *in vitro* and *in vivo*. *Anticancer Res.*, **13**, 801–806.

CITRON, M., DECKER, R., CHEN, S., SCHNEIDER, S., GRAVER, M., KLEYNERMAN, L., KAHN, L.B., WHITE, A., SCHOENHAUS, M. & YAROSH, D. (1991). O$^6$-methylguanine-DNA methyltransferase in human normal and tumor tissue from brain, lung and ovary. *Cancer Res.*, **51**, 4131–4134.

COHEN, A. & LEUNG, C. (1986). O$^6$-Methylguanine-DNA methyltransferase activity and sensitivity to N-methyl-N'-nitro-nitrosoguanidine during human T-lymphocyte differentiation. *Carcinogenesis*, **7**, 1877–1879.

COLVIN, M. & CHABNER, B.A. (1990). Alkylating agents. In *Cancer Chemotherapy Principles and Practice*, Chabner, B.A. & Collins, J.M. (eds), pp. 276–314. Lippincott: Philadelphia.

DEN ENGELSE, L., VAN BENTHEM, J. & SCHERER, E. (1990). Immunocytochemical analysis of *in vivo* DNA modification. *Mutat. Res.*, **233**, 265–287.

D'AMBROSIO, S.M., WANI, G., SAMUEL, M., GIBSON-D'AMBROSIO, R.E. & WANI, A.A. (1990). Repair of O$^6$-methylguanine damage in normal human tissues. IN *DNA Damage and Repair in Human Tissues*, Sutherland, B.M. & Woodhead, A. (eds), pp. 397–416. Plenum Press: New York.

D'INCALCI, M., CITTI, L., TAVERNA, P. & CAPITANO, C.V. (1988). Importance of the DNA repair enzyme O$^6$-alkylguanine alkyltransferase (AT) in cancer chemotherapy. *Cancer Treat. Rev.*, **15**, 279–292.

DOLAN, M.E., NORBECK, L., CLYDE, C., HORA, N.K., ERICKSON, L.C. & PEGG, A.E. (1989). Expression of mammalian O$^6$-alkylguanine-DNA alkyltransferase in a cell line sensitive to alkylating agents. *Carcinogenesis*, **10**, 1613–1619.

DOLAN, M.E., MOSCHEL, R.C. & PEGG, A.E. (1990). Depletion of mammalian O$^6$-alkylguanine-DNA alkyltransferase activity by O$^6$-benzylguanine provides a means to evaluate the role of this protein in protection against carcinogenic and therapeutic alkylating agents. *Proc. Natl Acad. Sci. USA*, **87**, 5368–5372.

DOLAN, M.E., MITCHELL, R.B., MUMMERT, C., MOSCHEL, R.C. & PEGG, A.E. (1991). Effect of O$^6$-benzylguanine analogues on sensitivity of human tumor cells to the cytotoxic effects of alkylating agents. *Cancer Res.*, **51**, 3367–3372.

EBERLE, G. (1989). Ph D Dissertation, University of Essen, Germany.

EPSTEIN, R.J. (1990). Drug-induced DNA damage and tumor chemosensitivity. *J. Clin. Oncol.*, **8**, 2062–2084.

FORNACE, Jr, A.J., PAPATHANASIOU, M.A., HOLLANDER, M.C. & YAROSH, D.B. (1990). Expression of the O$^6$-methylguanine-DNA methyltransferase gene MGMT in MER$^+$ and MER$^-$ human tumor cells. *Cancer Res.*, **50**, 7908–7911.

FRANKFURT, O.S., SECKINGER, D. & SUGARBAKER, E.V. (1990). Flow cytometric analysis of DNA damage and repair in the cells resistant to alkylating agents. *Cancer Res.*, **50**, 4453–4457.

GERSON, S.L. (1989). Modulation of human lymphocyte O$^6$-alkylguanine-DNA alkyltransferase by streptozotocin *in vivo*. *Cancer Res.*, **49**, 3134–3138.

GERSON, S.L. & TREY, J.E. (1988). Modulation of nitrosourea resistance in myeloid leukemias. *Blood*, **71**, 1487–1494.

GERSON, S.L., TREY, J.E., MILLER, K. & BERGER, N.A. (1986). Comparison of O$^6$-alkylguanine-DNA alkyltransferase activity based on cellular DNA content in human, rat and mouse tissues. *Carcinogenesis*, **7**, 745–749.

GERSON, S.L., TREY, J.E. & MILLER, K. (1988). Potentiation of nitrosourea cytotoxicity in human leukemic cells by inactivation of O$^6$-alkylguanine-DNA alkyltransferase. *Cancer Res.*, **48**, 1521–1527.

GODFREY, D.B., BOUFFLER, S.D., MUSK, S.R.R., RAMAN, M.J. & JOHNSON, R.T. (1992). Mammalian cells share a common pathway for the relief of DNA replication arrest by O$^6$-alkylguanine, incorporated 6-thioguanine and UV photoproducts. *Mutat. Res.*, **274**, 225–235.

GONZAGA, P.E., POTTER, P.M., NIU, T., YU, D., LUDLUM, D.B., RAFFERTY, J.A., MARGISON, G.P. & BRENT, T.P. (1992). Identification of the cross-link between human O$^6$-methylguanine-DNA methyltransferase and chloroethylnitrosourea-treated DNA. *Cancer Res.*, **52**, 6052–6058.

HANSSON, J., KEYSE, S.M., LINDAHL, T. & WOOD, R.D. (1991). DNA excision repair in cell extracts from human cell lines exhibiting hypersensitivity to DNA-damaging agents. *Cancer Res.*, **51**, 3384–3390.

HARRIS, A.L., KARRAN, P. & LINDAHL, T. (1983). O$^6$-methylguanine-DNA methyltransferase of human lymphoid cells: structural and kinetic properties and absence in repair-deficient cells. *Cancer Res.*, **43**, 3247–3252.

HOCHLEITNER, K., THOMALE, J., NIKITIN, A.YU. & RAJEWSKY, M.F. (1991). Monoclonal antibody-based, selective isolation of DNA fragments containing an alkylated base to be quantified in defined gene sequences. *Nucleic Acids Res.*, **19**, 4467–4472.

KARRAN, P. & BIGNAMI, M. (1992). Self-destruction and tolerance in resistance of mammalian cells to alkylation damage. *Nucleic Acids Res.*, **20**, 2933–2940.

KYRTOPOULOS, S.A., AMPATZI, P., DAVARIS, P., HARITOPOULOS, N. & GOLEMATIS, B. (1990). Studies in gastric carcinogenesis. IV. O$^6$-methylguanine and its repair in normal and atrophic biopsy specimens of human gastric mucosa. Correlation of O$^6$-alkylguanine-DNA alkyltransferase activities in gastric mucosa and circulating lymphocytes. *Carcinogenesis*, **11**, 431–436.

LEDOUX, S.P., THANGADA, M., BOHR, V.A. & WILSON, G.L. (1991). Heterogeneous repair of methylnitrosourea-induced alkali-labile sites in different DNA sequences. *Cancer Res.*, **51**, 775–779.

LEE, S.M., THATCHER, N. & MARGISON, G.P. (1991). O$^6$-Alkyguanine–DNA alkyltransferase depletion and regeneration in human peripheral lymphocytes following decarbazine and fotemustine. *Cancer Res.*, **51**, 619–623.

MITCHELL, R.B., MOSCHEL, R.C. & DOLAN, M.E. (1992). Effect of O$^6$-benzylguanine on the sensitivity of human tumor xenografts to 1,3-bis(3-chloroethyl)-1-nitrosourea and on DNA interstrand cross-link formation. *Cancer Res.*, **52**, 1171–1175.

MORIMOTO, K., DOLAN, M.E., SCICCHITANO, D. & PEGG, A.E. (1985). Repair of O$^6$-propylguanine and O$^6$-butylguanine in DNA by O$^6$-alkylguanine-DNA alkyltransferase from rat liver and *E. coli*. *Carcinoma*, **6**, 1027–1031.

MÜLLER, M.R., THOMALE, J., LENSING, C., RAJEWSKY, M.F. & SEEBER, S. (1993). Chemosensitisation to alkylating agents by pentoxifylline, O$^6$-benzylguanine and ethacrynic acid in haematological malignancies. *Anticancer Res.*, **13**, 2155–2160.

NEHLS, P. & RAJEWSKY, M.F. (1990). Monoclonal antibody-based immunoassay for the determination of cellular enzymatic activity for repair of specific carcinogen-DNA adducts (O$^6$-alkylguanine). *Carcinogensis*, **11**, 81–87.

PANELLA, T.J., SMITH, D.C., SCHOLD, S.C., ROGERS, M.P., WINER, E.P., FINE, R.L., CRAWFORD, J., HERNDON, II, J.E. & TRUMP, D.L. (1992). Modulation of O$^6$-alkylguanine-DNA alkyltransferase-mediated carmustine resistance using streptozotocin: a phase I trial. *Cancer Res.*, **52**, 2456–2459.

PEGG, A.E. (1990). Mammalian O$^6$-alkylguanine-DNA alkyltransferase: regulation and importance in response to alkylating carcinogens and therapeutic agents. *Cancer Res.*, **50**, 6119–6129.

PEGG, A.E., ROBERFROID, M., VON BAHR, C., FOOTE, R.S., MITRA, S., BRESIL, H., LIKHACHEV, A. & MONTESANO, R. (1982). Removal of O$^6$-methylguanine from DNA by human liver fractions. *Proc. Natl Acad. Sci. USA*, **79**, 5162–5165.

PIEPER, R.O., FUTSCHER, B.W., DONG, Q. & ERICKSON, L.C. (1991). Effects of streptozotocin/bis-chlorethylnitrosourea combination therapy on O$^6$-methylguanine DNA methyltransferase activity and mRNA levels in HT-29 cells *in vitro*. *Cancer Res.*, **51**, 2092–2097.

REDMOND, S.M.S., JONCOURT, F., BUSER, K., ZIEMIECKI, A., ALTERMATT, H.-J., FEY, M., MARGISON, G. & CERNY, T. (1991). Assessment of P-glycoprotein, glutathione-based detoxifying enzymes and O$^6$-alkylguanine-DNA alkyltransferase as potential indicators of constitutive drug resistance in human colorectal tumours. *Cancer Res.*, **51**, 2092–2097.

ROSS, G. & BROWN, R. (1992). The role of DNA repair processes in determining response to cancer therapy. *Eur. J. Cancer*, **28**, 281–285.

SAFFHILL, R. (1984). *In vitro* reaction of N-n-butyl-N-nitrosourea and n-butyl methanesulphonate with guanine and thymine bases of DNA. *Carcinogenesis*, **5**, 621–625.

SAGHER, D., KARRISON, T., SCHWARTZ, J.L., LARSON, R., MEIER, P. & STRAUSS, B. (1988). Low $O^6$-alkylguanine DNA alkyltransferase activity in the peripheral blood lymphocytes of patients with therapy-related acute nonlymphocytic leukaemia. *Cancer Res.*, **48**, 3084–3089.

SAMSON, L., THOMALE, J. & RAJEWSKY, M.F. (1988). Alternative pathways for the *in vivo* repair of $O^6$-alkylguanine and $O^4$-alkylthymine in *E. coli*: the adaptive response and nucelotide excision repair. *EMBO J.*, **7**, 2261–2267.

SCHERER, E., VAN DEN BERG, T., VERMEULEN, E., WINTERWERP, H.H.K. & DEN ENGELSE, L. (1989). Immunocytochemical analysis of $O^6$-alkylguanine shows tissue specific formation in and removal from esophageal and liver DNA in rats treated with methylbenzylnitrosamine, dimethylnitrosamine, diethylnitrosamine and ethylnitrosourea. *Cancer Lett.*, **46**, 21–29.

SEILER, F., KIRSTEIN, U., EBERLE, G., HOCHLEITNER, K. & RAJEWSKY, M.F. (1993). Quantification of specific DNA O-alkylation products in individual cells by monoclonal antibodies and digital imaging of intensified nuclear fluorescence. *Carcinogensis*, **9**, 1907–1931.

SIBGHAT-ULLAH & DAY, III, R.S. (1992). Incision at $O^6$-Methylguanine: Thymine mispairs in DNA by extracts of human cells. *Biochemistry*, **31**, 7998–8008.

SILBER, J.R., BOBOLA, M.S., EWERS, T.G., MURAMOTO, M. & BERGER, M.S. (1992). $O^6$-alkylguanine DNA-alkyltransferase is not a major determinant of sensitivity to 1,3-bis(2-chloroethyl)-1-nitrosourea in four medulloblastoma cell lines. *Oncol. Res.*, **4**, 241–248.

SOULIOTIS, V.L., BOUSSIOTIS, V.A., PANGALIS, G.A. & KYRTOPOULOS, S.A. (1991). *In vivo* formation and repair of $O^6$-methylguanine in human leukocyte DNA after intraveneous exposure to dacarbazine. *Carcinogenesis*, **12**, 285–288.

STRAUSS, B.S. (1990). The control of $O^6$-methylguanine-DNA methyltransferase (MGMT) activity in mammalian cells: a premolecular view. *Mutat. Res.*, **233**, 139–150.

THOMALE, J., HOCHLEITNER, K. & RAJEWSKY, M.F. (1993). Differential formation and repair of the mutagenic DNA-alkylation product $O^6$-ethylguanine in transcribed and non-transcribed genes of the rat. *J. Biol. Chem.* (in press).

VÄHÄKANGAS, K., TRIVERS, G.E., PLUMMER, S., HAYES, R.B., KROKAN, H., ROWE, M., SWARTZ, R.P., YAEGER, H. & HARRIS, C.C. (1991). $O^6$-Methylguanine-DNA methyltransferase and uracil DNA glycosilase in human broncho-alveolar lavage cells and peripheral blood mononuclear cells from tobacco smokers and non-smokers. *Carcinogenesis*, **12**, 1389–1394.

VAN DELFT, J.H.M., VAN WEERT, E.J.M., SCHELLEKENS, M.M., CLAASSEN, E. & BAAN, R.A. (1991). The isolation of monoclonal antibodies selected for the detection of imidazole ring-opened N7-ethylguanine in purified DNA and in cells *in situ*. Crossreaction with methyl, 2-hydroxyethyl and sulphur mustard adducts. *Carcinogenesis*, **12**, 1041–1049.

WALKER, M.C., MASTERS, J.R.W. & MARGISON, G.P. (1992). $O^6$-alkylguanine-DNA alkyltransferase activity and nitrosourea sensitivity in human cancer cell lines. *Br. J. Cancer*, **66**, 840–843.

WANI, G., WANI, A.A. & D'AMBROSIO, S.M. (1993). Cell type-specific expression of the $O^6$-alkylguanine-DNA alkyltransferase gene in normal human liver tissues as revealed by *in situ* hybridization. *Carcinogenesis*, **14**, 737–741.

ZHEN, W., LINK, C.J., O'CONNOR, P.M., REED, E., PARKER, R., HOWELL, S.B. & BOHR, V.A. (1992). Increased gene specific repair of cisplatin interstrand cross-links in cisplatin-resistant human ovarian cancer cell lines. *Mol. Cell Biol.*, **12**, 3689–3698.